# Clinical outcomes of pediatric macular edema associated with non-infectious uveitis

**DOI:** 10.1186/s12348-021-00236-4

**Published:** 2021-03-15

**Authors:** Anh Hong Nguyen, Bethlehem Mekonnen, Eric Kim, Nisha R. Acharya

**Affiliations:** 1 Department of Ophthalmology, University of California, 490 Illinois St, 2nd floor, San Francisco, CA 94158 USA; 2 Francis I. Proctor Foundation, University of California, San Francisco, USA; 3 Department of Epidemiology and Biostatistics, University of California, San Francisco, USA

**Keywords:** Pediatric uveitis, Macular edema, OCT

## Abstract

**Background:**

Macular edema (ME) is the most frequent cause of irreversible visual impairment in patients with uveitis. To date, little data exists about the clinical course of ME in pediatric patients. A retrospective, observational study was performed to examine the visual and macular thickness outcomes of ME associated with chronic, noninfectious uveitis in pediatric patients.

**Methods:**

Pediatric patients with noninfectious uveitis complicated by ME seen in the University of California San Francisco Health System from 2012 to 2018 were identified using ICD-9 and ICD-10 codes. Data were collected from medical records including demographics, diagnoses, ocular history, OCT imaging findings, complications, and treatments at first encounter and at 3, 6, 9, and 12-month follow-up visits. Cox proportional hazards regression was used to investigate the association between different classes of treatment (steroid drops, steroid injections, oral steroids and other immunosuppressive therapies) and resolution of macular edema.

**Results:**

The cohort comprised of 21 children (26 eyes) with a mean age of 10.5 years (SD 3.3). Undifferentiated uveitis was the most common diagnosis, affecting 19 eyes (73.1%). The majority of observed macular edema was unilateral (16 patients, 76.2%) and 5 patients had bilateral macular edema. The mean duration of follow-up at UCSF was 35.3 months (SD 25.7).

By 12 months, 18 eyes (69.2%) had achieved resolution of ME. The median time to resolution was 3 months (IQR 3–6 months). Median best-corrected visual acuity (BCVA) at baseline was 0.54 logMAR (Snellen 20/69, IQR 20/40 to 20/200). Median BCVA at 12 months was 0.1 logMAR (Snellen 20/25, IQR 20/20 to 20/50) Corticosteroid injections were associated with a 4.0-fold higher rate of macular edema resolution (95% CI 1.3–12.2, *P* = 0.01).

**Conclusions:**

Although only 15% of the pediatric patients with uveitis in the study cohort had ME, it is clinically important to conduct OCTs to detect ME in this population. Treatment resulted in 69% of eyes achieving resolution of ME by 12 months, accompanied with improvement in visual acuity. Corticosteroid injections were significantly associated with resolution of macular edema.

## Introduction

Treatment of children with uveitis presents unique challenges. Pediatric patients account for less than 15% of patients in most uveitis clinics [7]. Hence, limited research has focused on their clinical course. Uveitis in children is often asymptomatic and detected at an advanced stage with severe complications [1]. There is also some evidence that uveitis in children tends to be more treatment-resistant, with inflammation more likely to be recurrent or chronic and having a higher likelihood of vision loss [1, 7]. Thirty-four percent of children with uveitis present with at least one ocular complication at the time of diagnosis and 86.3% have ocular complications by 3 years after diagnosis [1]. These complications, such as cataracts, glaucoma, posterior synechiae and macular edema, often lead to irreversible structural damage and significant visual disability [1, 4].

Macular edema is the most common cause of vision loss in adults with uveitis, and the few prior studies conducted in pediatric patients have shown macular edema to be significantly associated with moderate and severe vision loss [7]. Although the incidence of ME in children appears to be lower than adults, 8% of legal blindness that results from pediatric uveitis is attributable to macular edema [2].

Given the potentially severe impact of macular edema on vision in pediatric patients and the limited information available, this study aimed to assess clinical outcomes of macular edema in children with noninfectious uveitis and determine how treatment affects visual and macular thickness outcomes.

## Methods

This was a retrospective study of pediatric patients with noninfectious uveitis and macular edema conducted using the electronic health record system at the University of California San Francisco Medical Center, including the Francis I. Proctor Foundation. This study was approved by the UCSF Institutional Review Board and followed the tenets of the Declaration of Helsinki.

Patients were included if they were below 18 years of age, had a diagnosis of non-infectious uveitis with macular edema, and had an Optical Coherence Tomography (OCT) showing macular edema. Two hundred thirty-one patients with uveitis between 2012 and 2018 were identified by performing an electronic query for ICD 9 and 10 codes in patients 18 years of age or younger. Electronic record review was performed to identify patients with non-infectious uveitis who had macular edema documented by OCT. Macular edema was defined by a central macular thickness of greater than 320 μm by Heidelberg spectral-domain OCT with or without the presence of intraretinal cysts. As a result, 34 of 231 (14.8%) pediatric uveitis patients were identified as having non-infectious uveitis with macular edema. Twenty-one of these patients had at least 6 months of follow-up and good quality macular OCTs. Patients with a follow-up course shorter than 6 months, poor quality or no macular OCT, infectious causes of uveitis, and macular edema related to retinal vascular diseases were excluded (i.e. familial exudative vitreoretinopathy, choroidal neovascular membrane secondary to uveitis, and Coats Disease).

If a patient was found to be eligible, the following data were recorded: demographic information (age, race/ethnicity, sex), systemic diagnosis, Snellen best-corrected visual acuity, duration of macular edema (when able to be determined), duration of uveitis, treatment history, uveitis activity (anterior chamber cells and vitreous haze according to the Standardization of Uveitis Nomenclature criteria), macular central subfield thickness, type of macular edema (cystoid, diffuse or sub-retinal) and lens status.

Participants’ Snellen best-corrected visual acuity, central subfield thickness, uveitis activity, treatments and ocular complications were recorded from the following 4 follow-up time points: 26 eyes (100%) at baseline, 26 eyes (100%) at 3 months, 26 eyes (100%) at 6 months, 24 eyes (92%) at 9 months, and 24 eyes (92%) at 12 months after the first encounter for uveitic macular edema at the UCSF clinic. The closest follow-up visit within 2 weeks of each time point was used. Lea Symbols were used to obtain Snellen best-corrected visual acuity in children not well-versed in the alphabet. Of note, some patients were already diagnosed with macular edema at first presentation to UCSF. Not all data were consistently available at every time point in the patient records, resulting in a different number of eyes for some variables. Additionally, fewer OCT data were available at later time periods, since clinicians likely did not measure central macular thickness many months after macular edema resolution. Macular edema was deemed to have resolved when central macular thickness was less than or equal to 320 μm and no intra-retinal cysts were identified.

Statistical analysis was performed using R, version 3.6.1 (R Project for Statistical Computing). Descriptive statistics on continuous data were presented in the form of means and SDs, or medians and interquartile ranges (IQR). Snellen visual acuity was converted to logMAR for analysis. A Cox proportional hazards model was used to calculate the hazard ratio of macular edema resolution associated with various treatments (topical steroids, oral steroids, steroid injection/implants, and systemic immunomodulatory therapies/biologic therapies). Patients could have been on one or more therapies. Treatment was coded as a dichotomous variable at the eye level (ever received a particular treatment vs never during the treatment course for ME). A hazard ratio of greater than 1 means that a treatment is correlated with a higher rate of ME resolution. A Chi-square test for homogeneity was performed to assess the correlation between categorical variables and macular edema resolution.

## Results

The study cohort consisted of 26 eyes in 21 children, of which 13 were female and 8 were male. The mean age was 10.5 years (SD 3.3). Sixteen patients (76.2%) had unilateral macular edema (Table 1). Twenty-two eyes out of 26 eyes (84.6%) had active uveitis at the time of initial presentation of ME at UCSF. The average duration of follow-up was 35.3 months (SD 25.7).

**Table 1:** Demographic Characteristics of Patients with Non-infectious Uveitic Macular Edema (*n* = 21)

**Age**	Mean 10.5 years, SD 3.3	
**Follow-Up**	Mean # of follow-up visits	27.5, SD 18
	Mean months of follow-up at UCSF	35.3, SD 25.7
		**# of people (%)**
**Sex**	Male	8 (38.0%)
	Female	13 (62.0%)
**Laterality of uveitis**	Unilateral	5 (23.8%)
	Bilateral	16 (76.2%)
**Race**	Caucasian	4 (19.0%)
	Hispanic/Latino	6 (28.6%)
	African-American	2 (9.5%)
	Asian	2 (9.5%)
	Unknown	7 (33.3%)

The mean duration of uveitis at the time of ME diagnosis was 9.6 months (SD 11.2). Nineteen eyes (73.1%) had undifferentiated uveitis, 4 eyes (15.4%) had pars planitis, and 3 eyes (11.5%) had juvenile idiopathic arthritis-associated uveitis. Four eyes (15.3%) had anterior uveitis, 14 eyes (53.8%) had anterior/intermediate uveitis, 2 eyes (7.7%) had intermediate uveitis, and 6 eyes (23.1%) had panuveitis (Table 2). There was no association between the anatomical designation of uveitis and rate of macular edema resolution (*P* = 0.28). The morphology of the macular edema was cystoid in 9 eyes (34.6%), cystoid/diffuse in 5 eyes (19.2%), cystoid/sub-retinal fluid in 2 eyes (7.7%), diffuse in 6 eyes (23.1%), diffuse/sub-retinal fluid in 1 eye (3.8%) and unknown morphology in 3 eyes (11.5%). There was also no association between macular edema morphology and rate of resolution (Table 2, *P* = 0.16).

**Table 2:** Uveitis and Macular Edema Characteristics (*n* = 26 eyes)

		# of eyes (%)
**Anatomic Designation**	Anterior	4 (15.4%)
	Anterior-Intermediate	14 (53.8%)
	Intermediate	2 (7.7%)
	Panuveitis	6 (23.1%)
**Uveitis Diagnosis**	Idiopathic	19 (73.1%)
	Pars Planitis	4 (15.4%)
	JIA-associated	3 (11.5%)
**Complications at baseline**	Cataract	9 (34.6%)
	Band Keratopathy	5 (19.2%)
	Reduced visual acuity that may be due to amblyopia	5 (19.2%)
	Posterior Synechiae	3 (11.5%)
	Retinal Vasculitis	3 (11.5%)
	Glaucoma	1 (3.8%)
**Morphology of Macular Edema**	Cystoid	9 (34.6%)
	Cystoid/diffuse	5 (19.2%)
	Cystoid/sub-retinal fluid	2 (7.7%)
	Diffuse	6 (23.1%)
	Diffuse/sub-retinal fluid	1 (3.8%)
	Unknown	3 (11.5%)

Patients presented with one or more complications at baseline

The most common pre-existing ocular complications were cataracts (9 eyes, 34.6%), band keratopathy (5 eyes, 19.2%), posterior synechiae (3 eyes, 11.5%), retinal vasculitis (3 eyes, 11.5%), glaucoma (1 eye, 3.8%), and reduced visual acuity prior to diagnosis of ME that may be due amblyopia (5 eyes, 19.2%) (Table 2). Some patients had a history of ocular surgery: 2 eyes (7.7%) with a history of cataract surgery, 2 eyes (7.7%) with retinal surgery, and 1 eye (3.8%) having undergone surgery for glaucoma.

**Fig. 1:**
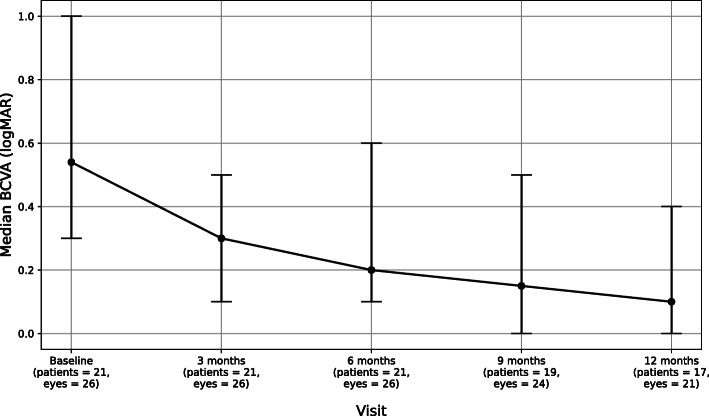
Median Best-Corrected Visual Acuity (BCVA) over 12 Months. A decrease in logMAR corresponds to an improvement in visual acuity. Two patients were lost to follow-after the 6 months visit, resulting in 19 patients (24 eyes) at the 9 months mark. At the 12 month mark, no additional patients were lost to follow-up but 2 patients (1 unilateral, 1 bilateral, 3 eyes) did not have their visual acuity recorded in the medical records at that visit

Best-corrected visual acuity (BCVA) at baseline had a median of 0.5 logMAR (IQR 0.3–1.0, *n* = 26 eyes) (Snellen 20/69, IQR 20/40 to 20/200). The median BCVA subsequently improved to 0.3 logMAR at 3 months (IQR 0.1–0.5, n = 26 eyes) (Snellen 20/40, IQR 20/25 to 20/69), 0.2 logMAR at 6 months (IQR 0.1–0.6, n = 26 eyes) (Snellen 20/32, IQR 20/25 to 20/79), and 0.2 logMAR at 9 months (IQR 0–0.5, *n* = 24 eyes) (Snellen 20/28, IQR 20/20 to 20/69). At the final 12-month follow-up visit, the median visual acuity was 0.1 logMAR (IQR 0–0.4, *n* = 21) (Snellen 20/25, IQR 20/20 to 20/50) (Table 3, Fig. 1).

**Table 3:** Median Visual Acuity and Central Macular Thickness (CMT) Over 12 Months Follow-Up

Outcome	Baseline	3 Months	6 Months	9 Months	12 Months
**Median BCVA in logMAR**	0.54 (IQR 0.3–1.0, *n* = 26)	0.30 (IQR 0.1–0.5, *n* = 26)	0.20 (IQR 0.1–0.6, *n* = 26)	0.15 (IQR 0–0.5, *n* = 24)	0.1 (IQR 0–0.4, *n* = 21)
**Median BCVA in Snellen**	20/69, IQR 20/40 to 20/200	20/40, IQR 20/25 to 20/69	20/32, IQR 20/25 to 20/79	20/28, IQR 20/20 to 20/69	20/25, IQR 20/20 to 20/50
**CMT (microns)**	424 (IQR 352–620.5, *n* = 23)	325 (IQR 283–347, *n* = 21)	310 (IQR 298–325, *n* = 22)	305 (IQR 285–319, *n* = 19)	302 (IQR 281–340, *n* = 18)

**Fig. 2:**
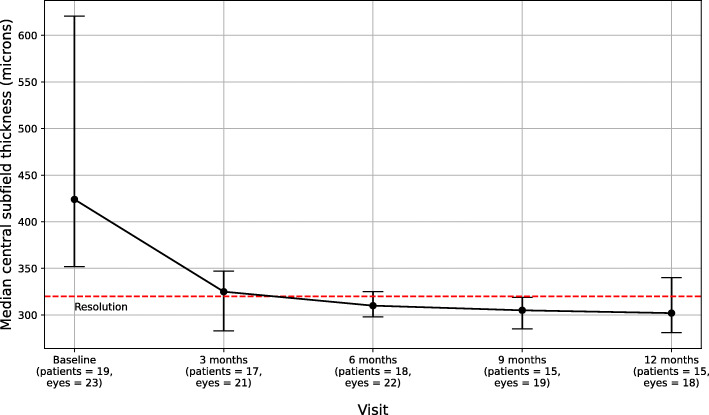
Central macular thickness changes over 12 Months. 55.6% achieved macular edema resolution by the 3-month mark. Two patients (1 with bilateral uveitis, 1 with unilateral) did not have OCT imaging at their baseline visit but were noted in their medical record to have macular edema from OCTs done by the referring clinic. Given that this is a retrospective chart review study, OCT data availability varied at different timepoints depending on the whether the provider ordered OCTs based on their clinical suspicion of macular edema

The median central macular thickness (CMT) decreased from 424 μm at baseline (IQR 352–620.5, *n* = 23 eyes) to 302 μm at 12 months (IQR 281.3–340.5, *n* = 18 eyes). The largest decrease in median CMT had occurred by the 3rd month, to 325 μm (IQR 283–347, n = 21 eyes) (Table 3, Fig. 2). Eighteen of the 26 original eyes (69.2%) achieved resolution of their macular edema by the 12-month point. Among the eyes that achieved resolution, the median time to resolution of ME was 3 months (IQR 3–6). Ten (55.6%) of the 18 eyes with resolution of ME were free of ME by the 3-month visit. Six more eyes resolved by the 6-month visit and 2 more were free of ME by the 9th month of observation.

Treatment regimens varied by patient and over time, with some patients switching treatment over the duration of this study. Furthermore, some patients were treated with multiple types of medications simultaneously. Of the 26 eyes, 23 (88%) were treated with biologics or immunosuppressive treatment (i.e. antimetabolites) at some point in their treatment. A Cox proportional hazards multivariable model adjusting for each of the treatments found the following hazard ratios (HR) for macular edema resolution: biologics or immunosuppressive treatment (23 eyes, 88.5%) HR 2.81 (95% CI 0.4–21.2, *P*-value 0.32), oral prednisone (11 eyes, 42%) HR 0.76 (95% CI 0.3–2, *P*-value 0.56), topical steroid drops (15 eyes, 58%) HR 0.52 (95% CI 0.2–1.3, *P*-value 0.16) and intravitreal steroids injections/implants (5 eyes, 19%) HR 4.0 (95% CI 1.3–12.2, *P*-value 0.01). Patients who were ever treated with steroid injections had a significantly higher rate of ME resolution. The remaining types of treatment did not have a significant association with macular edema resolution.

## Discussion

Among pediatric patients with non-infectious uveitis, the prevalence of macular edema was 15%. The patients in this study were treated in a tertiary care uveitis clinic where OCTs are routinely conducted in patients with uveitis, including children. The clinic has a standard imaging protocol where every new patient in uveitis clinic gets an OCT. After that, if there are macular issues, OCT is repeated on follow-up visits to track progress and even monitor after resolution. If the macula is normal on the initial visit, OCT is repeated on follow-up visits if there are other signs of active inflammation on exam, any reduction in visual acuity, or concern about macular abnormality on clinical exam. Given this, 15% is likely an accurate estimate of the prevalence of uveitic macular edema in pediatric uveitis patients. Although the percentage of affected pediatric patients is small, it is important to point out that those who were identified and treated experienced a clinically meaningful improvement in visual acuity. Almost 40% of patients who received treatment achieved resolution by the 3-month follow-up date. Most of the gains in VA were achieved by 3 months of follow-up to a median BCVA of 20/45. Fourteen out of 21 eyes (66.7%) achieved ≥20/40 BCVA by the 12-month follow-up date.

The results of this study are similar to prior retrospective studies that demonstrated favorable visual outcomes in pediatric patients with macular edema of various non-infectious etiologies ( [3, 6], .and [5].) In the series by Eiger-Moscovich et al. of 25 children (33 eyes), 75.8% achieved resolution of macular edema by 24 months. Patients in their series had similar uveitis etiologies and also received a varied combination of treatment regimens, including a combination of local corticosteroid injections or dexamethasone implants, systemic corticosteroids, antimetabolites and anti-TNF alpha agents. They found no association between outcome and any specific treatment strategy. This study had limited power to assess the impact of specific treatments but did find that steroid injections had a significant association with resolution of macular edema and systemic immunosuppressive therapy demonstrated a signal towards being beneficial.

With regard to the etiology of macular edema, this study did not find any difference in outcomes across the various anatomic designations of uveitis. Similarly, this study did not show any correlation between morphology of macular edema on OCT and macular edema resolution. These findings are largely in line with that of Eiger-Moscovich et al. Their study suggested a non-significant trend towards faster resolution of macular edema in patients with subretinal fluid.

Given the tertiary referral setting, reliably determining duration of macular edema was not possible since some patients had ME on their initial referral to UCSF that was not previously diagnosed. Pediatric patients also present a unique challenge in monitoring, given difficulties in cooperation on obtaining OCT evaluation and varying practices in frequency of macular edema screening. These factors often lead to a delay in diagnosis [2] and difficulty in determining macular edema duration, a limitation of this study.

This study faced other limitations. Despite beginning the review with 231 records, the study resulted in a small sample size due to the rarity of non-infectious uveitis complicated by macular edema in the pediatric population. This issue is further compounded by inconsistency in OCT and visual acuity measurements across follow-up visits even in patients who did follow-up. These unfortunately are the limitations of a retrospective study. Secondly, although treatment data was collected, the lack of standardized treatment meant that many patients were on multiple and varying combinations of treatments, making it difficult to ascertain the effectiveness of any particular treatment. This area would benefit from further research to determine the effectiveness of single and combination therapies.

To date, there have been a limited number of studies on macular edema among pediatric patients with non-infectious uveitis. This study augments the literature by reporting on the clinical course of macular edema among 21 children in a tertiary care setting and demonstrates that treatment is associated with improvement in macular edema and visual acuity. In conclusion, even though the percentage of pediatric patients with non-infectious uveitis complicated by macular edema is relatively low, the findings suggest that OCTs for detection of macular edema are important in this population since treatment can have a clinically meaningful impact.

## Data Availability

The dataset used and analyzed during the current study is available from the corresponding author on reasonable request. The data is not publicly available to protect patient privacy.

## Abbreviations

BCVA: Best-corrected visual acuity
CMT: Central macular thickness
HR: Hazard ratio
IQR: Interquartile range
ME: Macular edema
OCT: Optical coherence tomography
SD: Standard deviation
UCSF: University of California, San Francisco
